# Effects of a standardized patient-based simulation in anaphylactic shock management for new graduate nurses

**DOI:** 10.1186/s12912-022-00995-y

**Published:** 2022-08-01

**Authors:** Qi Ren, Fang Chen, Huijuan Zhang, Juanhua Tu, Xiaowei Xu, Caixia Liu

**Affiliations:** 1 Intensive care unit, Zhejiang Hospital, Hangzhou, Zhejiang China; 2 Nursing department, Zhejiang Hospital, No. 1229 Gudun Road, Zhejiang Hangzhou, China; 3 Cardiovascular ward, Zhejiang Hospital, Hangzhou, Zhejiang China; 4 Gynecology ward, Zhejiang Hospital, Hangzhou, Zhejiang China

**Keywords:** New graduate nurses, Anaphylactic shock, Standardized patient-based simulation, Simulation training

## Abstract

**Background:**

Patients may be endangered if new graduate nurses cannot recognize and manage anaphylactic shock. Consequently, enhancing the new graduate nurses’ understanding of their roles and responsibilities during the rescue of a patient with anaphylactic shock is important. However, due to its inherent limitations, traditional classroom-based teaching makes it difficult to explore the potential of the students. Although popular simulation teaching has several notable advantages, it has not been proven to be effective in training inexperienced nurses on anaphylactic shock. We investigated the effect of a standardized patient-based simulation on the behaviors of new graduate nurses’ during anaphylactic shock rescue to identify an effective and safe method for contemporary nursing education.

**Methods:**

Except for the ill or pregnant, all the new graduate nurses were included in the study as students to undergo a standardized patient-based simulation conducted in the clinical skills center of a general hospital. The simulation training was designed to teach students to recognize the signs and symptoms of anaphylactic shock, place the patient in the correct position, stop the ongoing intravenous infusion of the antibiotic which triggers the anaphylactic shock, restart an intravenous infusion on a new infusion apparatus, give 100% oxygen via a nasal cannula or mask, preserve airway patency, call the rapid response team, and correctly administer the medications prescribed by the clinicians. Before and after the training, the instructors evaluated each student’s skills and behaviors using a clinical competency evaluation list. After the training, all students completed the Chinese version of the *Simulation Design Scale* (SDS) to demonstrate their satisfaction with the program and then participated in semi-structured interviews with their instructors.

**Results:**

All 104 graduate nurses had a significant improvement on the 6 competencies of the clinical competency evaluation list after the simulation training (*P* < 0.001). The SDS scores revealed that the students were highly satisfied with all the aspects of the simulation training (the 20 satisfaction rates were all above 90.00%). During the semi-structured interviews, most of the new graduate nurses reported that simulation training in the management of anaphylactic shock was critical and would guide them in clinical practice.

**Conclusion:**

Simulation training in anaphylactic shock is a potentially viable and effective method for teaching new graduate nurses to manage clinical incidents.

## Background

Anaphylactic shock is a severe, potentially fatal hypersensitivity reaction that necessitates immediate medical attention [1]. It can be triggered by a variety of antigens including the venom of some insects and reptiles, antibiotics, nonsteroidal anti-inflammatory drugs (NSAIDs), glucocorticoids, opiates, vaccines, foods, as well as physical factors such as cold and exercise [2–4]. Anaphylactic shock is one of the most prevalent diseases in emergency departments and ICUs globally. Over the last few decades, the prevalence of allergic diseases in developed countries has been increasing steadily [5]. Anaphylactic shock progresses rapidly and can result in serious complications such as cardiac arrest [6]. Therefore, inexperienced new graduate nurses may be overwhelmed and fail to rescue patients with anaphylactic shock. Errors by nurses who cannot recognize the signs and symptoms, as well as manage anaphylactic shock can endanger the patients [7, 8]. Hence, there is a need for educational interventions to increase knowledge of anaphylactic shock in new graduate nurses.

Traditional classroom-based teaching is insufficient to prepare nurses to deliver safe and competent professional care for patients with anaphylactic shock [9]. Studies suggest that simulation training, a popular teaching method in the current medical field, can improve nurses’ clinical knowledge and performance in detecting and responding to clinical emergencies [10–15]. Standardized patient-based simulation is one of the primary means of simulation training and a teaching method with some simulated scenarios or cases by standardized patients (SPs). The approach can replicate real clinical situations, prompting students to solve clinical problems as well as make key decisions. The novel method has been demonstrated to be effective in the management of chronic disease, evaluation of pharmacology proficiency, training of communication skills, and treatment of postpartum hemorrhage, among others [16–19]. However, in most Chinese nursing schools, opportunities to participate in simulation training particularly standardized patient-based simulation are scarce. Thus, most new graduate nurses have little experience to deal with time-critical situations, like anaphylactic shock. Nursing lecturers must assess the effectiveness and safety of standardized patient-based simulation methods for contemporary nursing education, especially for inexperienced nurses. This study described a simulated scenario of anaphylactic shock that enabled new graduate nurses to assess and manage patients with critical anaphylactic shock without putting the patient at risk. Findings should be used in the development of teaching methods that facilitate the early identification and management of anaphylactic shock and improve the clinical competency of new graduate nurses.

## Methods

### Design, participants, and ethical considerations

This study used convenience sampling of 104 students and 10 instructors in quasi-experimental research. All the students had to be new graduate nurses contracted to work for one year in our hospital in Zhejiang province, China. They had no prior simulation experience. Nurses who were unable to participate in the training due to illness or pregnancy were excluded from this research. All 104 new graduate nurses were fully aware of the study and volunteered to participate. The study was approved by the ethics committee of our hospital. All the instructors were clinical nurses in our hospital with over 10 years of clinical nursing experience and over 3 years of clinical preceptor experience. After receiving onsite training from the professors of the University of Pittsburgh, they were awarded the simulation-based teaching certificates of the *Improving Simulation Instructional Methods* (ISIM) program developed at this university. To ensure the homogeneity of training, the selected instructors had an identical length of work and teaching experience at the time. They concentrated on lesson preparation and unified lesson plan 2 days in advance.

A total of 10 volunteers were recruited as SPs, all of whom were clinical nurses in our hospital. The training for these 10 SPs was mainly divided into basic knowledge training and script reading. The basic knowledge training was mainly to review the medical theory knowledge involved in the script and to fully understand the concept, responsibility, significance, and requirements of an SP. It also encompassed the introduction of performance skills. Script reading required the SPs to understand the script, correctly describe the history and symptoms and make consistent body language and expressions. The instructors would first demonstrate, and then the volunteers copied, performing in front of a mirror to correct their movements. The vital signs of these 10 SPs were all vital signs generated by the teaching ECG monitor in their beds. The instructors had completely control over the changes in vital signs, which were read by the students via computer programs.

### Procedure

The simulation training program was an integral part of the new graduate nurse’s 2-year standardization training. Every new graduate nurse should complete the compulsory credits of this program to train and demonstrate their emergency rescue competence. A rescue protocol training checklist for anaphylactic shock developed with advice from the professors at the University of Pittsburgh was used in this training. Its content was designed to teach students to recognize the signs and symptoms of anaphylactic shock, place the patient in the correct position, stop the ongoing intravenous infusion of the antibiotic which triggers the anaphylactic shock, restart an intravenous infusion on a new infusion apparatus, give 100% oxygen via a nasal cannula or mask, preserve airway patency, call the rapid response team (RRT), and correctly administer the medications prescribed by the clinician (Table 1).

**Table 1:** Implementation of the program of simulation-based training (The rescue protocol training checklist for anaphylactic shock)

Phase	Content	Time
1. Introduce the simulation	Training questions	1 min
2. Recognize allergic shock	Nursing assessment	2 min
3. Position training	Correct positioning of a patient with allergic shock	1 min
4. Call the RRT	Familiar with the RRT’s number and can call it correctly	1 min
5. Oxygen	Openning the airway and giving oxygen about 4–6 L/min	2 min
6. Medications	Steroids are given per MD’s order	2 min
7. Debriefing	Training feedback	10 min

All the students were randomly assigned to 1 of 10 groups, each having 1 instructor and 10 to 14 students. Before the training, they were introduced to the principles of managing anaphylactic shock by the pre-briefing in the classroom. The instructors conducted one on one simulation training with each student immediately after the in-class session for the program of simulation training. The training on the basis of the rescue protocol training checklist for anaphylactic shock lasted for about 20 minutes.

The simulation training was held in our hospital’s clinical skills center and the primary site were 10 simulated wards which were all equipped with a bed, a bedside table, a chair, an SP, and clinical supplies including a nasal cannula, an oxygen tubing, a mask, a bag valve, a treatment cart, an intravenous pump, a peripheral IV set, and some injection needles. They would also contain drugs such as dexamethasone, normal saline, and Ringer’s solution. 

The simulation room resembled a cardiac ICU. The scenario introduced to the learners was: “The patient is 68 years old, male, and has a history of hypertension and coronary heart disease. He had been admitted to the hospital due to dizziness and chest pain. The environment was safe for both the patient and the nurse. You were his charge nurse, and when the patient was not feeling well, he was to ring for you.” The patient had chest pain, sweating, and purple lips 10 minutes after intravenous antibiotic. His vital signs were as follows: heart rate was 95 beats per minute, blood pressure was 60/40 mmHg, and body temperature was 36℃. The patient was flushed, delirious, and gasping for breath. Their lower extremities were not swollen.

The students have assured safety in the simulation-provided environment in which they could gain clinical experience. The objective for the students was to stop the antibiotic intravenous infusion, restart an intravenous infusion of normal saline, give the patient oxygen, maintain the airway, call the RRT, and administer steroids as prescribed by the clinician. The instructors observed whether the students correctly followed the rescue procedure for anaphylactic shock.

Following the training, the students were allowed to review their simulation experience through instructor-guided debriefings using the “Gather, analysis, and summarize format (GAS)”. The debriefing began with a brief description of the anaphylactic shock and accompanying rescue protocol to gather information and ensure all the students had a shared mental picture of the events. The students were asked: “Can you recall what you have done, what do you think?”. The anaphylactic shock rescue protocol was then analyzed using a “Plus-delta” method, in which the students reflected on what went well (plus) and what could have been improved (delta). For example, one of the instructor-guided questions could be “I noticed that you opened the airway, why?” and “Apart from calling bedside doctors, who else do you need to notify, and how?” The students responded with “I noticed that the patient having difficulty in breathing and was concerned about a possible swollen pharynx.” and “I should call the RRT immediately.” The debriefing concluded with students’ summarizing the key points learned and discussing how they will improve their future performance in anaphylactic shock scenarios.

A month after the 1st simulation training, the students underwent the process again. The case was still anaphylactic shock due to medications. The main symptoms and signs were the same as the first time, but their order of occurrence and degree were different, controlled by the instructors and SPs. The primary diseases of the SPs were also different. The instructors tracked the progress of the students through the program and recorded important data such as the change in their clinical competency.

### Measurements

#### Clinical competency evaluation list of the rescue for anaphylactic shock

The instructors employed a clinical competency evaluation list to assess every student’s skills and behaviors derived from the program of simulation training. The evaluation list included 6 competencies (assessment skills and ability to place the patient in a correct position, call the RRT, maintain an airway, administer oxygen and other therapies), each of which was scored “Yes” or “No” by the instructor. The results of the evaluation list in the 2 simulation trainings (the interval was one month ) were recorded and compared.

#### Learner feedback

##### **The Chinese version of the*Jeffries Simulation Design Scale*(SDS)**

The Chinese version of the *Jeffries Simulation Design Scale* (SDS) was translated by Zhu FF. et al. [20]. The scale is reliable (its total Cronbach’s *ɑ* was 0.948) and valid (its content validity index was above 0.830). The scale was specifically designed to evaluate a simulation training, optimize teaching programs, and improve the quality of teaching. Following the simulation training, the students were given the SDS to access quantitative data about the simulation training. The instrument had 20 items that assessed the features of the simulation training from 5 dimensions: (1) Objectives/information, (2) Support, (3) Problem solving, (4) Feedback, and (5) Fidelity. These items were rated on a 5-point Likert scale ranging from “Strongly disagree (1)” to “Strongly agree (5)”. The satisfaction of the students with the training was indicated by the satisfaction rate of each item. The satisfaction rate was calculated as the number of students who chose 4 and 5 divided by the total number of students. The average scores for each of the 5 dimensions in the 104 new graduate nurses were also calculated.

### Semi-structured interviews

Half an hour after the training, at the training site, the instructors in each group conducted semi-structured interviews with all the students in their group to get feedback on the program. Among the questions were: What have you gained from this training? What are your thoughts about this training? After assessing your performance during this training, which aspects did perform well and what could be improved in the future? Based on what you have learned from this program, will you adjust your care during your clinical practice, and will you integrate what you have learned into your daily clinical practice? As the students responded, the instructor recorded. The teacher then integrated and categorized the content of the recording to form several conclusions, and calculated the proportion of each conclusion among the students.

### Data analysis

To assure the correctness of data entry, Epidata 3.1 software was used to create a database, and double-entry verification was done. Statistical analysis was done in SPSS 22.0. Measurement data was reported as (*x* ± *sd*). Count data were reported as percentages. McNemar’s test was used for the comparison of the data before and after the training at *P* < 0.05.

## Results

The students consisted of 104 new graduate nurses (100 females and 4 males) with a mean age of 21.91 ± 2.05 years (range 21–28 years). 13 students had an Associate’s degree in nursing, 90 students had a Bachelor’s degree in nursing, and 1 student had a Master’s degree in nursing.

### Clinical competency evaluation list of the rescue for anaphylactic shock

After the 1st training, all the students significant improved in 6 competencies (assessment skills and ability to place the patient in a correct position, calling the RRT, maintaining an airway, administering oxygen and other therapies) assessed on the clinical competency evaluation list of the rescue for anaphylactic shock in the 2nd training (*P* < 0.001) (Table 2).

**Table 2:** The outcomes of the clinical competency evaluation list of the rescue for anaphylactic shock in the 1st and 2nd the training (*n* = 104)

Items	Outcomes of the accuracy				*P*-value of McNemar’s test
Learners’ assessment skills	In the 1st training	In the 2^nd^ training		Total	**< 0.001**
		Yes	No		
	Yes	68	0	68	
	No	34	2	36	
	Total	102	2	104	
Calling the RRT	In the 1st training	In the 2^nd^ training		Total	**< 0.001**
		Yes	No		
	Yes	75	0	75	
	No	28	1	29	
	Total	103	1	104	
Maintaining patents’ airway	In the 1st training	In the 2^nd^ training		Total	**< 0.001**
		Yes	No		
	Yes	41	0	41	
	No	61	2	63	
	Total	102	2	104	
Shock position	In the 1st training	In the 2^nd^ training		Total	**< 0.001**
		Yes	No		
	Yes	58	1	59	
	No	41	4	45	
	Total	99	5	104	
Administration of oxygen	In the 1st training	In the 2^nd^ training		Total	**< 0.001**
		Yes	No		
	Yes	31	3	34	
	No	55	15	70	
	Total	86	18	104	
Administration of medications	In the 1st training	In the 2^nd^ training		Total	**< 0.001**
		Yes	No		
	Yes	40	0	40	
	No	60	4	64	
	Total	100	4	104	

The evaluation list addressed six competencies, each of which was scored ‘Yes’ or ‘No’ by the instructors

### Learners’ feedback

After the training, all the students completed the Chinese version of SDS on time and reported their experiences through semi-structured interviews with their instructors. The SDS results revealed that the students were highly satisfied with all aspects of the training (Table 3).

**Table 3:** The students’ feedback on simulation design characteristics: the Chinese version of the *Jeffries Simulation Design Scale* (*n* = 104)

Dimensions	Items	1	2	3	4	5	Satisfaction rate (%)	The scores for each of the 5 dimensions (*n* = 104)
Objectives/information	I had adequate pre-training preparation and I was encouraged to participate.	0	0	2	7	95	98.08	24.49 ± 1.18
	I was provided clear and definite teaching goals.	0	0	1	5	98	99.04	
	During the simulation teaching, I was provided enough clinical information to facilitate me to solve the clinical problems.	0	0	3	4	97	97.12	
	I was provided with enough clinical information during simulation training.	0	1	2	2	99	97.12	
	The examples during teaching training have prepared me to understand simulation training.	0	2	4	2	96	94.23	
Support	I was provided adequate support and help during the simulation training.	0	0	2	1	101	98.08	19.78 ± 0.59
	Teachers can identify my needs when I need them.	0	0	2	2	100	98.08	
	Teachers have been very helpful during the simulation training.	0	0	1	2	101	99.04	
	I had all the support during my entire training.	0	0	2	4	98	98.08	
Problem-solving	My problem-solving skills have improved after this simulation training.	0	0	0	6	98	100	24.58 ± 0.78
	I was encouraged to search all the possibilities for solving problems during this simulation training.	0	1	3	5	95	96.15	
	This simulation training was designed according to my knowledge and clinical skills.	0	0	2	2	100	98.08	
	Simulation teaching has provided opportunities for me to improve my clinical assessment and nursing skills.	0	0	3	4	97	97.12	
	Simulation training has provided me with constructive feedback upon making clinical nursing goals.	0	0	2	4	98	98.08	
Feedback	Structured and well-organized feedback.	0	0	0	5	99	100	19.76 ± 0.51
	I was giving feedback promptly during simulation training.	0	0	2	5	97	98.08	
	I was allowed to analyze my clinical performance during the feedback session.	0	0	1	3	100	99.04	
	I was given feedback from training preceptors after simulation teaching finished.	0	0	0	6	98	100	
Fidelity	This simulation training was imitated by a real clinical environment.	0	1	3	2	98	96.15	9.84 ± 0.52
	This simulation training is real to me.	0	0	1	4	99	99.04	

Items were rated on a five-point scale ranging from “Strongly disagree (1)” to “Strongly agree (5)”

The students’ reflections on their experience during the semi-structured interviews demonstrated that they all believed that simulation training in the management of anaphylactic shock is important for new graduate nurses. Hence, 103 (99.04%) students reported that the simulation training would guide them in clinical practice. All the students stated that instructors taught them how to safely handle some emergencies and resuscitate patients. 98 (94.23%) students felt more knowledgeable about activating RRT calls. 96 (92.30%) students reported improvements in their clinical judgment and emergency response. 80 (76.92%) students acknowledged that there was still room for improvement in some areas such as opening the airway and administration of the correct medications. When compared to in-class training, 78 (74.29%) students reported that the simulation training enhanced their clinical judgment and emergency response successfully, and hoped to participate in more simulation training.

## Discussion

Nurses must make prompt decisions in the face of a critical incident, such as a patient in anaphylactic shock, despite the complex and dynamic circumstances [21]. They must have situational awareness and automation to perceive important information, comprehend present events, project future events, and avoid patient harm caused by preventable errors [22]. The current study describes a simulated scenario of anaphylactic shock. In this scenario, new graduate nurses were allowed to examine and manage a critically ill patient who had anaphylaxis in a safe environment that did not put a patient at risk. Following the simulation, the students reported a better understanding of their roles and responsibilities during the rescue of a patient with anaphylactic shock. This was consistent with the results of Agogo’s study on undergraduate medical students by using SPs [18]. Consistent with these findings, healthcare providers in a pediatric emergency unit provided simulation training in the management of anaphylaxis by using a high-fidelity patient simulator mannequin, and the training improved the ability to use epinephrine and refer patients to the allergy unit for evaluation [23], while nurses participating in clinical simulations of anaphylaxis in a hospital setting reported enhanced confidence in patient assessment skills, recognition of a problem, and accurate communication of findings to others on the healthcare team [24].

So why was it possible to derive positive learning outcomes through SP-based simulation, and what are the strengths of this study in terms of education, research and practice? The training concentrated on the simulation of the real scene which can strengthen the memory of the student, and provided a simulated bedside rescue scene for the new graduate nurses. Thus, the new graduate nurses can “personally visit” the real clinical practice and deal with it practically. Furthermore, the simulation training has strict steps and time arrangements. The course is coherent and compact, with each stage having its own predetermined time from the opening introduction to final feedback. The new graduate nurses realized their mistakes and were extremely impressed by the feedback of the course’s final phase, directed by the GAS. They were able to apply that knowledge in subsequent clinical work.

Simulation training provides learner-centered educational opportunities that are designed to demonstrate procedures in an environment that mimics the reality of a clinical setting. An instructor can use simulation to control the learning environment, provide immediate feedback, and promote students’ decision-making and critical thinking skills. Simulation training provides a relatively secure teaching setting in which students can learn from their mistakes, and develop the capacity to accomplish tasks [25, 26]. Several studies have demonstrated that simulation training is superior to didactics and demonstration for teaching technical and nontechnical skills to healthcare workers [27–29].

In the current study, instructors’ assessment of the students’ clinical competency pre and post-simulation showed significant improvements in the students’ assessment skills and their ability to place the patient in the correct position, call the RRT, maintain an airway, and administer oxygen and other therapies. The students learned to recognize anaphylaxis, identify and remove the antibiotic, and prevent clinical decompensation in the patient. They also acknowledged the importance of promptly calling the RRT whose role is to intervene as early as possible in a deteriorating patient to avoid further, preventable critical events and so reduce the in-hospital morbidity and mortality [30]. Multidisciplinary RRT has been successfully integrated into many hospitals resulting in improved resuscitation rates, increased morale and empowerment among nurses, redistribution of workload for nurses (reducing neglect of non-acutely ill patients during emergencies), and immediate access to expert help [31, 32]. The students learned about the resuscitation steps, including giving oxygen by face mask and aiming for an oxygen saturation above 92%, and the treatment options for a patient with anaphylaxis, such as epinephrine, antihistamines, and steroids.

The simulation received very positive feedback from the students. Simulation training provides a realistic clinical experience for students, facilitating professional development and increasing their self-confidence as members of a practicing healthcare team [33, 34]. Most students believed that the simulation mimicked the clinical environment and improved their understanding of early, rapid recognition and treatment of anaphylactic shock. About 99.04% of the students stated that the instructors had been helpful, while 97.12% of the students reported that the instructors were supportive. This was especially critical because some students were overwhelmed and helpless when faced with a patient who had difficulty in breathing, even during simulation. Previous studies have shown that simulation trainings in nurses boosts confidence, makes students aware of parts of care that need to be addressed via intentional practice, increases nurses’ willingness to learn and convey their knowledge, and strengthens communication among team members [33, 35, 36].

This study had some limitations. Firstly, the sample size was not calculated before the study as a literature search revealed no similar research for a reference. Fortunately, all new graduate nurses contracted to work at the hospital for one year participated in this study. Secondly, the study had no control group. However, the study sought to improve the clinical competency of all the new graduate nurses, a pretest-posttest study design was adopted. Thirdly, self-reports from students were used to evaluate the program of the training. Despite the ease with which their self-reports were obtained, the approach is associated with response bias due to false reporting and variations in students’ capacity to interpret questions and their introspective ability. Forthly, while the clinical competency evaluation list is not a questionnaire, it still needs to be validated to some extent. The items based on those experts’ opinions may not fully assess the ability of nurses in the management of anaphylactic shock. Future research should validate this list with large sample size or replace it with an established evaluation tool. Fifthly, there are certain restrictions in the Chinese version of the SDS. Because the scale is not commonly used, its dependability may be called into doubt. A few items have factor loadings < 0.4, which may affect the validity. Moreover, because this scale was not used to compare two groups, its evaluation function was not properly reflected [20]. Finally, the scenario was not tested as a reliable simulation of anaphylactic shock. Nevertheless, the outcomes of this study suggest that this simulation is applicable for training new graduate nurses, it demonstrated that the new method may explore the potential of nurses and improve their clinical judgment on safety. It provides new options for clinical nursing education, and thus warrants further development.

## Conclusion

The simulation training which used a simulated scenario of anaphylaxis was practical and effective as a method to teach new graduate nurses to manage clinical incidents of anaphylactic shock. It can build confidence in new graduate nurses, facilitating the provision of safe patient-centered care. Because there are differences in teaching level, teaching equipment and students’ competency in various hospitals, multi-center randomized controlled trials should be as much as possible in the future to comfirm the effectiveness of this teaching method in the emergency treatment of anaphylactic shock.

## Data Availability

The data and materials of the present study are available from the corresponding author on reasonable request.

## Abbreviations

RRT: Rapid response team
SDS: Simulation Design Scale
NSAIDs: Nonsteroidal anti-inflammatory drugs
ISIM: Improving Simulation Instructional Methods
SP: Standardized patient
IV: Intravenous injection
ICU: Intensive care unit
GAS: Gather, Analysis, Summarize format
